# Haptic Spatial Configuration Learning in Deaf and Hearing Individuals

**DOI:** 10.1371/journal.pone.0061336

**Published:** 2013-04-11

**Authors:** Rick van Dijk, Astrid M. L. Kappers, Albert Postma

**Affiliations:** 1 HU University of Applied Sciences School of Sign Language Interpreting, Utrecht, The Netherlands; 2 Helmholtz Institute, Experimental Psychology, Utrecht University, Utrecht, The Netherlands; 3 Helmholtz Institute, Physics of Man, Utrecht University, Utrecht, The Netherlands; 4 VU University Amsterdam, Faculty of Human Movement Sciences, Amsterdam, The Netherlands; 5 Department of Neurology, University Medical Centre Utrecht, Utrecht, The Netherlands; Bielefeld University, Germany

## Abstract

The present study investigated haptic spatial configuration learning in deaf individuals, hearing sign language interpreters and hearing controls. In three trials, participants had to match ten shapes haptically to the cut-outs in a board as fast as possible. Deaf and hearing sign language users outperformed the hearing controls. A similar difference was observed for a rotated version of the board. The groups did not differ, however, on a free relocation trial. Though a significant sign language experience advantage was observed, comparison to results from a previous study testing the same task in a group of blind individuals showed it to be smaller than the advantage observed for the blind group. These results are discussed in terms of how sign language experience and sensory deprivation benefit haptic spatial configuration processing.

## Introduction

Our sense of active touch is crucially important for exploration of peripersonal space. We can find, locate and handle objects within reach without having to look at them. Haptic information allows us to make fairly good estimates of item numbers [1] and even a haptic pop-out effect may occur for free manual exploration [2].

Despite its clear behavioral relevance the question is whether we all are equally sensitive to haptic inputs. Peck and Cilders [3] developed an instrument to assess a person's preference for using touch information and observed considerable individual differences. Interestingly, Kalisch et al. [4] observed an age decline for both tactile acuity (two point threshold) and haptic object recognition. The latter though was most clearly correlated to overall cognitive ability. Dijkerman and de Haan [5] report that brain damage may lead to a variety of touch deficits, ranging from finger agnosia, to impaired tactile object recognition, and denial of ownership of a body part.

An important source of individual variation in haptic ability might follow from sensory deprivation. In a previous study [6], we tested congenitally and late blind individuals against sighted controls on the portable Tactual Performance Test (pTPT), which is part of the Halstead–Reitan Test Battery [7]. Participants were blindfolded and had to fit in 10 familiar shapes in the matching cut-outs in a board as quickly as possible. This procedure was repeated three times. The blind were found to be faster but learning curves were comparable. Rotation of the board did not alter the group differences. In a trial, in which the participants had to freely relocate the shapes in a board of the same size but without the cut-outs blind and sighted individuals performed similarly. We concluded that greater reliance on the haptic inputs in the blind could have stimulated a better sense of object handling by touch. Visual experience on the other hand could be useful for constructing a more explicit spatial representation of the object array. It should be noted that there was no group difference for free relocation in the Postma et al. [6] study. However the fact that the initial blind advantage had disappeared could be taken as an indication that visual experience does play some role here.

Whereas effects of blindness on tactile performance have been well documented (e.g., [8] [9] [10], [11] [12] [13]), effects of chronic auditory deprivation on touch have rarely been investigated. As such, the goal of the *present* study was to compare active touch in deaf individuals to that in hearing controls and hearing sign language users. We used a set-up similar to that of [6]. We explored in particular two possibilities. One is that deafness itself leads to a concentration of attention on the remaining sensory input channels. Hence we would expect better performance in the deaf group. A slightly different variant of this possibility is that this advantage is restricted to the free relocation trials, because of a generally better developed visuospatial sense in deaf individuals. In contrast to the view that deafness itself leads to a difference in active touch, the second possibility is that it is sign language usage which underlies more profound tactile skills. For other domains, sign language usage has been found to yield a positive effect. Emmorey, Kosslyn and Bellugi [14] showed better image generation and rotation performance by ASL (American Sign Language) signers (hearing and deaf) over non-signers. Pyers, Shusterman, Senghas, Spelke, and Emmorey [15] point out that sign languages in particular offer a way to represent spatial relations iconically. If this indeed is so, we would expect the sign language users to be better on our haptic tests as well, but no difference should exist between deaf and hearing signers.

In order to contrast the foregoing two possibilities we included two planned comparisons in our analyses: deaf against hearing persons and signers against non-signers. In addition, we compared results from the present study to that of our earlier work with blind individuals. This allows further insights in which alterations in the other sensory domains could particularly affect our sense of touch.

## Methods

### Ethics statement

This research was in accordance with the Declaration of Helsinki, and the protocol was deemed to be without psychological or medical risks, and to comply with good ethical standards, by the ethical advisory committee of the Faculty of Social and Behavioral Sciences at Utrecht University. The ethical advisory committee of the Faculty of Social and Behavioral Sciences at Utrecht University approved the protocol, including the consent procedure. All participants signed a letter of informed consent prior to the start of the experiment.

### Participants

Three groups of participants performed the experiment: 15 (7 female, 8 male) deaf persons with prelingual deafness and sign language as their first language, 16 (8 female, 8 male) hearing sign language interpreters with Dutch as their first language and 16 (8 female, 8 male) hearing control persons with no sign language experience. Two deaf participants were left-handed, all others were right-handed, as assessed by means of a questionnaire [16].

To be included in the deaf group, participants had to fulfill the following criteria:

1. hearing loss measured 90 dBHL or higher in the better ear;
2. congenitally deaf;
3. primary language Sign Language of the Netherlands (SLN);
4. attended educational programs for 15 years or more;
5. intelligence within the normal range according to the Raven Progressive Matrices [17].

One female of the original deaf group of 16 participants had a Raven score below the normal range, and therefore she was left out of the analyses. The average age of this group was 41.4 years (age range 16–66 years) and they had on average 16.6 years of education. The average percentile score on the Raven was 83.0.

The interpreters were required to have a Bachelor degree in Sign Language interpreting. This ensured that their level of sign language was at a near native level. The average age of this group was 38.4 years (age range 26–51 years) and they had on average 16.9 years of education and a Raven percentile score of 80.3. The control participants were associated with the school of Sign Language Interpreting of the University of Applied Sciences Utrecht, the Netherlands. They had no direct experience with or skills related to the use of Sign Language. Their average age was 44.8 years (age range 26–57 years) and they had on average 17.1 years of education. Their Raven percentile score was on average 89.1.

Statistical analyses confirmed that the groups did not differ significantly on age (*F*(2,44) = 1.389, *p* = 0.260) or years of education (*F*(2, 44) = 0.374, *p* = 0.69). For all remaining participants the Raven score was within the normal range.

All participants also performed a number of other experiments on the same day. These are beyond the scope of the current paper and are reported elsewhere (e.g. [18]).

### Apparatus and stimuli

**Figure 1:**
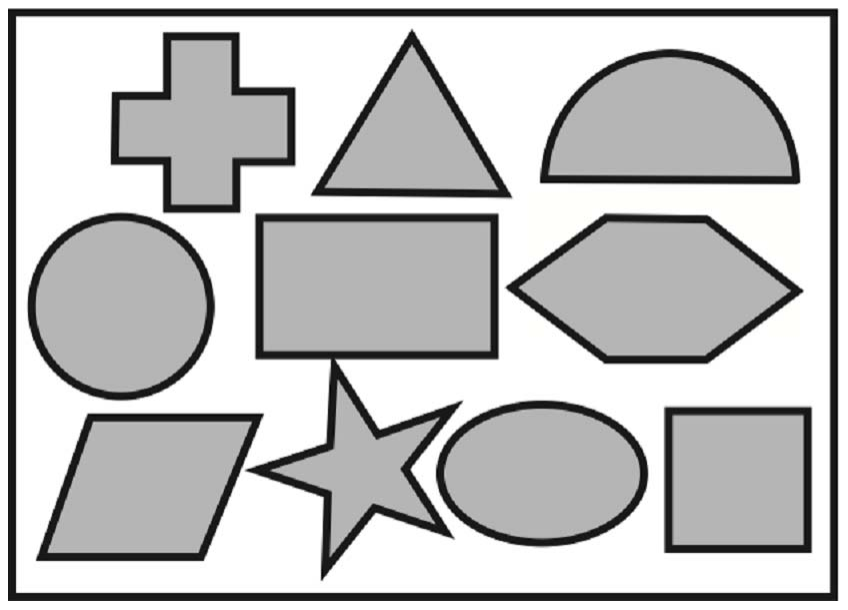
A schematic drawing of the objects and the board of the portable Tactual Performance Test (pTPT).

The same portable tactual performance test was used as in [6]. Figure 1 shows a schematic illustration. A wooden board of dimensions 45.5×30.2×2.1 cm contained ten shape cut-outs of ten geometrical objects: a cross, a triangle, a semicircle, a circle, a rectangle, a hexagon, a diamond, a star, an oval and a square. There was also a set of the ten geometrical objects that fitted exactly (and uniquely) in these cut-outs. This whole board was placed on a table in front of the blindfolded participant. For right-handed participants, the objects were placed on four piles on the right side of the board; for left-handed participants, these objects were placed to the left.

### Procedure

At the start and in between trials the wooden board was covered with a piece of paper, so that participants could never see the board or the objects. Just before the start of each trial they were informed what their specific task in that trial would be and subsequently they were blindfolded. In trials 1–3, they were asked to place the ten objects as fast as possible in the ten cut-outs. They were free in their choice of strategy and they were allowed to use both hands. The experimenter measured their exploration time by means of a stopwatch. Time started when participants first touched one of the shapes or the wooden board and ended when all objects were placed correctly in the cut-outs.

Just before trial 4, the wooden board was replaced by a wooden board of the same size, but with just a piece of paper instead of the cut-outs. Participants were asked to position the objects as accurately as possible in their original positions (that is, as if the cut-outs would have been there). In this trial, participants were allowed as much time as they preferred and time was not registered. After this trial, the experimenter traced the shapes with a pencil on the paper for later analysis.

In trial 5, the wooden board with the cut-outs was used again, but this time its orientation was rotated 90° counterclockwise. While the board was rotated, the participants hold their hands on the smaller sides, so that they also experienced how the board was rotated. Time was again recorded and they had perform this trial as fast as possible.

### Data analysis

#### General

As we were interested in the effect of both auditory and sign language experience, as well as possible interaction effects, we performed general Analyses of Variance (ANOVAs) with the three groups as a between-subject factor. Where necessary, the degrees of freedom were corrected by using Greenhouse-Geisser correction. Subsequently, we conducted two planned comparisons. In the analysis of auditory experience, we compared performance of the deaf participants with that of the interpreters and the controls. In the analysis of sign language experience, we compared performance of deaf participants and interpreters with that of the controls.

In trials 1–3 and 5, time to completion in seconds was the measure of performance. For trial 4, for each object, the deviation, that is the distance of the positioned object center from its correct position, was measured. The measure of performance was the average deviation over the ten different objects in cm.

#### Correlation analysis

It seems of interest to investigate the degree of learning, as observed in trials 1–3, with the performance in the free placement trial (4). As degree of learning percentage improvement was taken, defined as follows: 100%× (time in trial 1 - time in trial 3)/(time in trial 1). This measure was correlated with the average deviation per object observed in trial 4.

#### Comparison with blind participants

We have used the same task in a previous study with 13 congenitally blind (early blind), 17 late blind and 16 blindfolded sighted participants [6]. In that study, we found that blind participants (both early and late blind) outperformed blindfolded sighted participants. It is of clear interest to compare performance of the present groups with that of the groups of this earlier study. As completion times might be influenced by slight (unintended) changes in the procedure, the fair way to perform this comparison is to compute *Z*-scores with respect to the own control group. Therefore, the *Z*-scores were computed as follows:

*Z_i,j_* = (*t_i,j_*−<*t_j_*_-controls_>)/sd*_j_*_-controls,_ where *t* indicates the time to completion, *i* a specific participant, *j* a specific trial (1–3 or 5), <*t_j_*_-controls_> means the average time to completion for the controls in that same trial and, sd*_j_*_-controls_ gives the standard deviation over the control group in that trial.

## Results

### Time to completion

**Figure 2:**
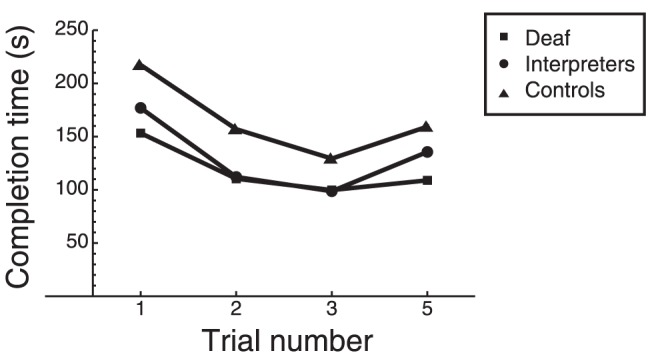
Completion times for the deaf (D), the interpreters and the controls. In trials 1–3 the board was oriented as shown in Fig.1, whereas in trial 5, the board was rotated 90° counterclockwise.

In Figure 2 the average completion times for trials 1–3 and 5 are shown for the three different groups. An ANOVA with trial as within-subject factor and group as between-subject factor showed a significant effect of trial (*F*(2.45, 107.68) = 20.24; *p*<0.001; η^2^ = .315). Repeated contrasts showed that trial 1 differed significantly from trial 2; trial 2 differed from trial 3, and trail 3 differed from trial 5. The group effect failed to reach significance (*F*(2, 44) = 2.57; *p* = 0.088). The interaction Trial by Group was not significant either.

Subsequently, two planned comparisons were performed. First, auditory experience (deaf versus interpreters and controls) was analysed. Although the effect of trials was again significant (*F*(2.45, 110.37) = 15.74, *p*<0.001; η^2^ = .259), there was no significant difference between the groups. Second, the use of sign language (deaf and interpreters versus controls) was analysed. Again the effect of trial was significant, *F*(2.44, 109.9) = 20.06; *p*<0.001; η^2^ = .308), but interestingly, also the difference between the groups turned out to be significant (*F*(1, 45) = 4.86, *p* = 0.033; η^2^ = .098): signers were faster than non-signers.

### Deviations

The average deviation of the ten geometrical objects was 11.6 cm for the deaf group, 10.1 cm for the interpreters and 10.1 cm for the controls. These values were not significantly different from each other.

### Correlations

**Figure 3:**
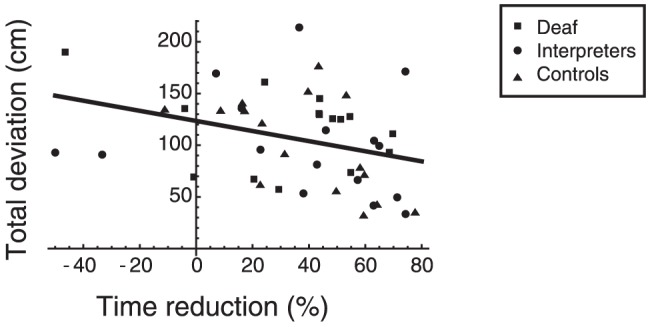
Correlation between the average deviation per object in cm measured in trial 4 and the percentage time reduction from trial 1 to trial 3.

Most participants improved performance (i.e., became faster) from trial 1 to trial 3. This improvement can be quantified by the percentagel time reduction. Figure 3 shows the average deviation per object in cm in trial 4 as a function of this time reduction from trial 1 to trial 3 for the three participant groups. The correlation between these two performance measures was significant (*r* = −0.33; *p* = 0.02). A relatively stronger learning rate as indicated by a larger percentage time reduction inspired smaller spatial deviations on the free replacement in trial 4.

### Comparison with the performance of blind observers

**Figure 4:**
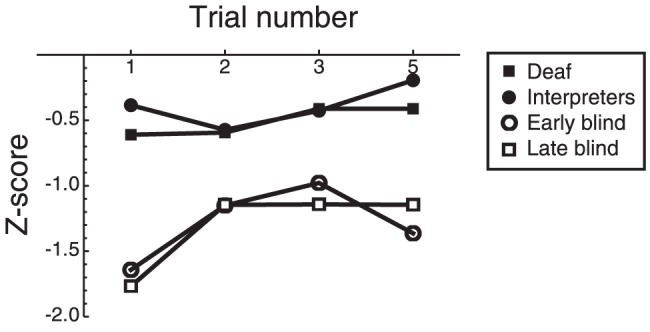
*Z*-scores for the deaf (D) and interpreters with respect to the controls from the current study compared to the *Z*-scores of the Early and Late blind observers with respect to their controls from the study by Postma et al. (2007).

Previously we have conducted a similar study with blind observers as participants [6] and observed better performance in the blind than in sighted controls. It is of clear interest to establish whether the current sign language user advantages are comparable to the blindness advantages in the Postma et al paper [6]. Figure 4 therefore shows the *Z*-scores of the four different groups (each computed with respect to its own control group). It can be seen that the Z-scores of the two blind participants groups were lower (i.e., more different from the control group) than those of the two other groups. An ANOVA with trial as within-subject factor and group as between-subject factor showed indeed a significant effect of group (*F*(3,57) = 7.34; *p*<0.001; η^2^ = .279). Also the effect of trial (*F*(3, 171) = 6.38; *p*<0.001; η^2^ = .101) and the interaction between trial and group (*F*(9, 171) = 2.27; *p* = 0.02; η^2^ = .107) were significant. Repeated contrasts showed that trial 1 differed from trial 2, but trial 2 did not differ from trial 3, nor did trial 3 differ from trial 5. The interaction group x trial was also significant for the contrast trial 1 vs trial 2, but not for any of the other contrasts. Together, this suggests that the relative difference between blind participants and controls (as indicated by the *z*-scores) is most notable in the first trial.

## Discussion

The goal of the present study was to compare active touch in deaf individuals to that in hearing controls and hearing sign language users. We employed a task assessing haptic object handling/exploration and spatial configuration learning. Blindfolded participants had to fill in object shapes in the corresponding slots of a rectangular board as quickly as possible. Participants were found to speed up with learning over trials. Interestingly, we observed that the deaf group did not differ from the hearing group (signers, controls) but the signing group (deaf and hearing interpreters) did outperform the non-signers (the hearing controls) on the first three learning trials as well as on trial 5 in which the board was rotated. The difference stayed stable over these trials. Hence, learning rate was comparable as well as haptic spatial updating. Most importantly, results indicate that it is not deafness itself which affects active touch ability, but rather it is the sign language experience.

Which elements of active touch mostly benefit from sign language usage? We may contrast haptic object handling/exploration against haptic spatial configuration learning. The fact that learning rates over the first three trials were similar for the three groups suggests that it is mostly the former. This notion is further confirmed by the fact that relocation scores in free space (i.e. within a board without slots) were also similar for the three groups. In the Introduction we mentioned the possibility that deaf persons might possess stronger visuospatial processing ability. This could have stimulated in particular relocation in the free space trial. In addition, Pyers et al. [15] pointed out that sign language usage ameliorates the construction of iconic spatial representations. Neither of these conjectures was supported in the present study. We think a different haptic spatial memory task might be more suitable to examine this. The board frame used here offered limited free space. Performance as such depended more strongly on relative position sense than on absolute ‘metric’ spatial memory. Future studies should try out different spatial memory and perception tests.

In [6] we speculated that the performance on the first three trials reflects the construction of a more implicit spatial representation, whereas free space relocation in trial 4 requires an explicit spatial representation. It is clear that the two levels of representation cannot be fully apart. If anything the processing of information in the beginning is a critical requirement for constructing a spatial map in trial 4. To test this connection we correlated the learning rate over the first three trials (i.e. the perceptual time reduction between trial 1 and 3) with the deviations in replacement in trial 4. A moderate, significant correlation was obtained. When the learning rate is stronger one is also better at relocating the shapes in free space.

The foregoing suggests that auditory deprivation may affect the processing of haptic information, though in an indirect way. Our previous work with blind individuals indicated that visual deprivation also has an impact on haptic processing in the current task. A comparison between the effect sizes showed a larger improvement in early and late blind individuals than in signing deaf and hearing individuals. Again this difference seems restricted to haptic handling of objects and object/space exploration, whereas it does not extend to the haptic spatial configuration learning.

In conclusion, the present study showed an advantage for both deaf and hearing sign language users for active touch in the exploration of peripersonal space. We argue that primarily the identification of objects and their placement in the corresponding slots may have benefited. The learning of the spatial display did not show any difference. The advantage seems directly linked to sign language experience. More research employing a larger variety of haptic spatial memory tasks is needed to further assess this issue. In comparison to previous work with blind individuals the observed advantages seem smaller. The question has often been raised which loss of sensory modality would have larger impact. With regard to the employment of the remaining haptic information channel in the present task our results indicate that the visual impairment effect surpasses that of auditory deprivation.
